# Narratives of neoliberalism: ‘clinical labour’ in context

**DOI:** 10.1136/medhum-2014-010606

**Published:** 2015

**Authors:** Bronwyn Parry

**Keywords:** Social science

## Abstract

Cross-border reproductive care has been thrust under the international spotlight by a series of recent scandals. These have prompted calls to develop more robust means of assessing the exploitative potential of such practices and the need for overarching and normative forms of national and international regulation. Allied theorisations of the emergence of forms of clinical labour have cast the outsourcing of reproductive services such as gamete donation and gestational surrogacy as artefacts of a wider neoliberalisation of service provision. These accounts share with many other narratives of neoliberalism a number of key assertions that relate to the presumed organisation of labour relations within this paradigm. This article critically engages with four assumptions implicit in these accounts: that clinical labourers constitute a largely homogeneous underclass of workers; that reproductive labour has been contractualised in ways that disembed it from wider social and communal relations; that contractualisation can provide protection for clinical labour lessening the need for formal regulatory oversight; and that the transnationalisation of reproductive service labour is largely unidirectional and characterised by a dynamic of provision in which ‘the rest’ services ‘the West’. Drawing on the first findings of a large-scale ethnographic research project into assisted reproduction in India I provide evidence to refute these assertions. In so doing the article demonstrates that while the outsourcing and contractualisation of reproductive labour may be embedded in a wider neoliberal paradigm these practices cannot be understood nor their impacts be fully assessed in isolation from their social and cultural contexts.

## Introduction

The recent baby Gammy furore^1–3^ has again focused attention on the practice of transnational or cross-border reproduction and specifically on outsourced gestational surrogacy (GS).^4^ ^i^ This and other high voltage events (such as the case of ‘stateless’ babies Manji Yamada and the twin Balaz children) have proven extremely strong catalysers of retroactive regulation in India^5^ and now also in Thailand, as evidenced by the military junta's prospective ban on all forms of commercial surrogacy.^6^ These reactionary responses produce profound ripple effects that reverberate out to all the other corners of the global reproductive marketplace. The tide of moral panic and restrictive regulation that drew commissioning parents away from Thailand now washes them up into the slew of newly funded fertility clinics in Mexico opened specifically to address their unmet needs.^ii^

The disturbing conditions of these particular transactional arrangements have prompted calls for GS to be either outlawed as an abject alienation and commodification of both bodily labour and the resultant child^7^ ^8^ or, alternatively, more highly formalised as a market through the development of international conventions or more consistent application of national regulations. Calls for the implementation of a global convention on transnational surrogacy (which might mirror the *Hague Convention on Transnational Adoption*, with presumably all its potential, as well as its proven shortcomings) have been sustained by the thesis that the practice remains inherently exploitative of the workers who perform such services, typically lower-income women in developing or middle-income countries.^iii^

Kirby's recent construction of a novel heuristic device for evaluating the exploitative potential of GS purports to provide a more dependable calculative tool for determining the relative costs and benefits of such forms of employment.^9^ Indeed, a crisp diagnosis that transnational GS in rural western India does meet the material conditions of exploitation is duly proffered following an analysis employing this methodology. However, what this article, Macer's response,^10^ and Orfali and Chiappori's^11^ accompanying commentary actually reveals is first, how dependent such tools are on presumptions about the commensurability of the kinds of labour and labour relations involved in such practices and second, how disparate these prove to be in practice. Any overarching analytical model or regulatory framework must be prepared to arrive at a normative position on the conditions under which such practices can or should be performed and yet, in so doing, risk expurgating the nuances of local lived experience that militate against the generation of a singular account or effective prognoses of how to secure ‘best practice’.

This said, unduly atomised accounts also risk obscuring the wider political economies of biological commodification that have emerged in post-Fordist economies in which these practices are undeniably embedded. Recent analyses have made valuable contributions to theorising how reproductive service work has become constituted as a form of economically productive ‘clinical labour’ in the neoliberal era. However, they also risk perpetuating some rather well-worn narratives about the impact of neoliberalism on reproductive service labour that obliterate key contextual factors, factors that actually create a much more complex geography of provision than such accounts presuppose. In this article, I draw upon preliminary findings from my Wellcome Trust funded study of the expansion of assisted reproduction in India^iv^ in order to trace some of the contradictions and nuances in experiences of contracted clinical labour that inevitably complicate attempts to unify them under a given sign, or for a specific governmental end, no matter how worthy.

## Characterising clinical labour

A range of recent literature has carefully explicated the ways in which the generative capacities of ‘life itself’ to employ Nikolas Rose's^12^ term, have been drawn into circuits of capital accumulation. These are constituted not only as alienated tissues, organs, reproductive cells and DNA, but also as forms of embodied labour (particularly reproductive and experimental labour) that have become key generators of value and drivers of innovation in the bioeconomy. Melinda Cooper and Catherine Waldby in their recently released book argue that the in vivo biology of human subjects is becoming increasingly central to the organisation of the post-Fordist economy, particularly the life science industries.^13^ As they note these industries increasingly rely ‘on an extensive, yet unacknowledged labour force, whose service consists in the visceral experience of experimental drug consumption, hormonal transformation, more or less invasive biomedical procedures, ejaculation, tissue extraction and gestation’—forms of work that they go on to characterise as ‘clinical labour’.^ii^

They argue, following Boltanski and Chiapello,^14^ that the emergence of post-Fordist models of economic organisation and governance has invoked profound shifts in the organisation of labour. We have moved from a mid-twentieth century system characterised, in the West at least, by statutory labour protections for industrial workers and secure conditions of employment, to a far more precarious, late twentieth century regime of labour. This typically involves outsourcing to private contractors with a concomitant increase in exposure to occupational health and safety risks and financial exploitation. Clinical labourers—those individuals who act as ‘independent contractors of their ‘biological capital’ are, they argue, even more tightly enmeshed in these new forms of precarious service labour, yet they, even more than even their historical counterparts, ‘labour without labour protection’, being ‘obliged to assume both the economic and corporeal risks of the biomedical innovation economy’. The key actors in the drama of clinical labour are here typically characterised, as they are elsewhere, as the ‘contingent workers of the bioeconomy’. These include, to use Cooper and Waldby's examples, those who engage in high risk phase I clinical trial work in exchange for money, uninsured patients who take part in clinical trials in exchange for medication, poorer women involved in egg vending, and those ‘donating’ organs and tissues to banks for money.^15^

What emerges very clearly from this narrative is the sense that these clinical labourers are, in many respects, victims of a voracious neoliberalism: exploited financially, in unstable outsourced employment, working under oppressive contractual service relationships in the bioeconomy. The gendered and racialised divisions of labour that attend the organisation of these new forms of bodily exploitation both echo and indeed amplify those that have historically underpinned the formalisation and commercialisation of other kinds of intimate relations in advanced economies including personal caregiving, child rearing, housekeeping and prostitution. These workers intersect, it is argued, ‘with the lowest echelons of informal service labour, being recruit[ed] from the same classes marginalised by the transition from Fordist mass manufacture to post-Fordist informatics production’.^iii^ As the sociologist Viviana Zelizer has noted, many social critics and scholars maintain that intimate relations and economic activity occupy distinct domains, the former ‘a sphere of sentiment and solidarity’, the latter ‘a sphere of calculation and efficiency’, contact between which, it is argued, produces moral contamination.^16^ This doctrine of antagonistic spheres motivates efforts to maintain a hygienic separation between the two. It particularly animates the concerns that attend the commercialisation of reproductive labour and the potential this is thought to have in corrupting the sacredness of the act of child bearing. It is often presumed, for example, that the exportation of this labour from the intimacy of the domestic space to the transactional floor of the biomedical clinic necessitates its detachment from the social and kin relationships of home and community with associated increases in physical and psychological harm.^17^

In constructing such arguments a number of assertions are made about the ways in which this new clinical reproductive labour force is constituted, contractualised, regulated and enrolled into wider transnational fertility markets and circuits of exchange. Clinical labourers are, within such accounts, usually characterised as an underclass that exist at the margins, oppressed, financially exploited and operating under precarious conditions of employment. Reproductive labour is said to be increasingly contractualised; removed from the space of the home and relocated within the more manageable and standardised confines of the clinic and consequently disembedded from the orbit of social and kin relations. Regulatory oversight is said to be generally resisted by the reproductive services market but contracts, when enacted, are thought to provide an adequate means of protection for outsourced labour. Reproductive labour is now also said to be increasingly transnationalised—however, the flow of labour and reproductive materials is assumed to be largely unidirectional, characterised by a dynamic of exploitation in which ‘the West exploits the rest’ (with attendant gender, class and racial politics). While there may be little contention over the broad thrust of these accounts, closer ethnographic work can serve to illuminate some very important distinctions and variations in practice within and between the different sectors and geographical loci of this emergent economy that serve to complicate this narrative. It is to an analysis of these that I now turn.

## Classes of clinical labour

There are, to my mind, several troubling aspects to these theorisations of the emergence of clinical reproductive labour. First among these is the tendency to conflate many, very distinct, types of labour and labourers under one rubric. This has the effect of erasing key aspects of their lived experience and of inviting the reader to assume that all clinical labourers occupy similar positions within the bioeconomy. Freighted with this comes a set of overarching normative assumptions about the kinds of labour these individuals contribute and most importantly their motivations for contributing that labour—a set of assumptions that, I would argue, prove to be rather context insensitive. In order to recalibrate this analysis I begin by examining the work of a particular class of reproductive labourers—gamete donors. As space is short I can only sketch out some of the key distinguishing features of the experiences of this highly variegated class of clinical labourers, to illustrate them I concentrate here on just a few: Californian sperm donors and Indian oocyte and sperm donors from Mumbai.

In many (but not all) respects, the women who agree to become oocyte donors in Mumbai are, as Waldby and Cooper surmise, among the most disadvantaged and poorly educated women in the city, although are certainly not unintelligent. Many are exceptionally capable and some are themselves very entrepreneurial. As others have noted, and as the findings from our research confirm, many of the women who agreed to act as donors (and later surrogates) were motivated to do so to alleviate either permanent or temporary states of indebtedness.^18^ ^19^ Unlike surrogates, the regulations do not demand that they be married and many of the recruited donors are young single women whose only alternative forms of work are among the lowliest in the service sector, including occupations such as vegetable vendor, domestic servant or piece workers. The same cannot be said of the Indian sperm donors we interviewed for this study. These men belonged to a much more advantaged social class. Unlike the oocyte donors, many are professionals, such as engineers or accountants, and they are not typically motivated to donate by financial need.

In this respect, they are actually more closely aligned both socially and in motivational terms with the Californian men who perform reproductive service labour for one of the world's largest sperm banks CaliforniaCryo. As I have noted elsewhere,^20^ the international market for US sperm is now in excess of 100 million dollars annually and California Cryo enjoys a 65% share of that export market. They consequently occupy a position right at the very heart of this global ‘bioeconomy’ in reproductive services. The first thing to know about this particular cohort of donors is that they are not just anyone. The bank proudly boasts that they accept <1% of all individuals that apply to become donors. Their criteria for inclusion reveal much about why the ‘failure’ rate is so high. The bank has a formal policy of rejecting any donor who is less than 5′9″ tall or who is not either currently undertaking a 4-year degree course at a University or who already has a bachelor or higher educational degree. They also note that their preference is to ‘actively recruit donors from top US universities’^21^ such as Harvard, Yale, Princeton and the like.

The bank considers these qualities to be simply ‘basic requirements’ for recruitment, noting that only those who meet them will be moved onto ‘the next step in our qualification process’. This involves, as they describe it: ‘conform[ing] to our unwavering benchmarks by which potential sperm donations are measured, which includes everything from extensive medical testing to genetic screening’. Far from being economically or socially marginalised these donors actually constitute *a biosocial elite:* exceptionally well educated, highly paid and comprised exclusively, to use selective breeding vernacular, of ‘high genetic merit individuals’. They do receive some minimal ‘compensation’ for their labour but they are not in any way dependent on that income. There is no financial compulsion for them to perform this labour.

These individuals, I would argue, would not construct themselves, nor could they feasibly be constructed as, exploited or oppressed clinical service workers. I would suggest, conversely that they perceive themselves to be benefactors of a kind of ‘philanthropic labour’, one animated by a concern for human welfare and advancement and manifested by the endowment of capital—in this case biological capital—to needy persons for ‘socially useful purposes’. Despite other differences in their life circumstances this is a motivation that they share with their Indian counterparts, many of whom conceptualise donation as a form of ‘social work’. To conflate the radically different experiences and motivations of these various reproductive workers is to risk erasing all the complexities that, in practice, complicate the generation of overarching narratives of neoliberalism and its effects.

## The contractualisation of reproductive labour

A defining characteristic of the neoliberalisation of reproduction is said to be its desequestration from the home and its associated reordering as a form of contracted and outsourced economic production. Capturing the value of reproductive labour efficiently demands, it is argued, that it be rationalised, an outcome that can be most readily achieved by disembedding it from the messy vagaries of communal life and relocating it within the controllable space of the clinic. In recent accounts of surrogacy in India much attention has thus been focussed on the ways in which spatial segregation has been employed as a tool to discipline women's potentially unruly reproductive labour. This is achieved it is said, through their relocation from their former communities to purpose built surrogate dormitories ‘where clinic staff control not only their health and nutritional status but even their abilities to freely interact with their families back home and the ‘intended parents’’.^22^ Associated with this is the presumption that risk (for non-compliance, failure to deliver, defectiveness and some others) can, and is, effectively displaced from the clinic to the surrogate through the adoption of that sanitary masculinised instrument: the private contract.

What all this implies is a clear formalisation of labour relations and demarcation of private and public roles and responsibilities in relation to the administration of this contract and the delivery of these reproductive services. What our recent ethnographic research in Mumbai has actually revealed is that these distinctions are, in practice, far from secure. The surrogates that we have interviewed have, technically, entered into a formal contract with the hospital clinic for whom they have agreed to act. However, they have not been *recruited* by the hospital or the staff there but rather through a very complex and extended network of social and communal relations and specifically by agents who are usually known to them. These may include male agents who have historically been involved in organising the distribution of piecework or other kinds of contractual labour or female agents. These are women who, having completed their own quotient of allowed surrogacies (two per married woman) have effectively begun to franchise their business by identifying young woman whose personal circumstances will predispose them to recruitment. These agents are not strangers to the potential recruit and despite the existence of the contract they do not have an employer–employee relationship.

On the contrary the agents’ success in recruitment is often attributable to their detailed personal knowledge of the prospective donor or surrogate's life circumstances and particularly the life crises (death of a relative, marital abandonment etc.) that can act to precipitate their participation. So tightly is this network of surrogates, agents and donors constituted in the communities in which we have conducted research in Mumbai, that we have yet to interview an agent, surrogate or donor who is unknown to another. Some of the relationships between individuals in the network are biological, others simply mirror extended familial and particularly maternal relations. It is not uncommon, for example, for young surrogates or egg donors to refer to their older female agents as Auntie (*Mausi*) or Sister (*Didi*), terms that are typically used to denote blood relationships but which are here employed to appeal to wider actual or imagined kinship obligations. Interestingly, although the agents could fall back on the device of the contract to limit the scope of these obligations^v^ they often don't, preferring instead to work outside the formal terms of the contract to provide additional food, medications, travel assistance or other others forms of support to secure the female donor or surrogates’ continued involvement. Even senior female medical staff in the clinics we observed provided additional undocumented assistance to surrogates and donors in times of need, actions that cemented personal and often maternal relationships of care that also extended well beyond anything required by the official terms of the contract.^vi^ These are the actual means through which these core relationships (on which the whole enterprise of outsourced reproductive labour rests) are built and sustained and risks managed. What this reveals is that it is not these workers imagined extraction from their social and kin relations that facilitates the commercialisation of their reproductive labour, but rather their very situatedness within them. While the outsourcing and contractualisation of reproductive labour may be embedded in a wider neo-liberal paradigm their underlying dynamics cannot be understood, I would argue, in isolation from their social and cultural contexts which, as this research suggests, dramatically shape their localised forms and practice.

## Outsourcing, regulation and enforcement

Outsourcing, the contractualisation of a business practice to a third party, externalises what would previously have been a social responsibility (the care and protection of the labour force) to the independent contractor in ways that appear to obviate the need for wider forms of regulatory oversight—such as labour or health and safety laws. Deregulation is considered a defining feature of neoliberal economics and is often identified as key causal factor in crises (such as the global financial crisis) said to be triggered by a lack of oversight. As economists Panich and Konings have argued ‘this diagnosis of the cause of the crisis also steers towards a particular solution: if deregulation allowed markets to get out of control, then we must look to re-regulation as the way out’.^24^ The transnationalisation of reproductive care, the associated and exponential increase in fertility service provision in India since 2010,^vii^ reports of malpractice and exploitation, and the long delay in the ratification of Indian's Draft *Assisted Reproductive Technologies (Regulation) Bill* (2010) have together worked to construct a similar narrative in this instance: that the expansion of the sector is both an artefact of the wider neoliberalisation of the Indian economy one characterised and facilitated by a laissez faire approach to regulation.^26^ The perceived remedy lies, predictably, in the institutionalisation of more formal regulations that, whether actually ratified or just imminent, would prompt more robust institutional protocols—and, in theory, stronger and more defensible contracts that better protect the interest of the clinical reproductive labourer.

Mirroring like arguments put forward by liberal feminist scholars in relation to the formalisation of regulations surrounding sex work, some advocates suggest that surrogates and oocyte donors should seek protection from exploitation by recourse to the same laws and mechanisms available to workers in other trades and occupations including, for example, via unionisation and the formulation of more comprehensive contracts that take greater account of their conditions of work.^27^ ^28^ INSTAR (The Indian Society for Third-Party Assisted Reproduction) which is comprised of a collective of infertility specialists, lawyers, embryologists and social workers from 15 Indian states, has recently lobbied for the implementation of more highly specified contracts for assisted reproduction. These would set minimum levels of compensation for reproductive services, and establish means of redress for a range of possible harms to donor, surrogate or intending parents, including for example in the event of miscarriage, ectopic pregnancy or birth defect.^29^ On the face of it these new protocols, which establish, for example, that a surrogate will receive full compensation after 28 weeks of gestation irrespective of the outcome of pregnancy, would generate enhanced protections that could, as Kirby argues, render the practice less exploitative.

A central tenet of the discourse around the deregulation of economic activity under neoliberalism is that corporations and institutions will vehemently resist the imposition of regulations as they delimit or constrain their abilities to act unitarily in profit maximising ways. This thesis has animated many analyses of the emergence of contractualised reproductive labour and infertility services in India and is promulgated in assertions that the sector is thus almost completely unregulated—that it is, as one colleague put it ‘like the wild West out there’. In distinct contrast to this our research has determined that most of the surrogacy and donation work that we witnessed was to all intents and purposes quite highly regulated. Detailed formal contracts were issued, signed and honoured. The senior staff of the hospitals and clinics that we interviewed made it clear to us that, contrary to public perception, they have no fear at all of regulation. In fact, they welcomed more robust forms of regulation and were perfectly willing to institute any revisions to contracts recommended by organisations such as INSTAR or which might later be demanded of them by the ratification of the new Assisted Reproductive Technologies (ART) bill.^viii^ Why is this?

There are several reasons. First, regulation in India acts as an extremely useful tool for streamlining surrogacy markets and resolving their ever present ‘crisis of legitimacy’. All of the emphasis in the contract is on remuneration and the payment of appropriate compensation for unforeseen contingencies. At present, the levels of remuneration that surrogates and donors in India receive remain very low in relation to those offered in more advanced economies. Money is therefore not a primary concern for hospitals and clinics. In fact, increasing remuneration provides a very low cost means of legitimating practices that have historically drawn wide disapprobation. Meeting demands for contractual refinements affords clinics and hospitals the opportunity to both perform and proclaim their conformance to a finite, and it would seem normative, set of ethical and legal standards. This lends their work credibility, deflects censure and creates an important selling point in national and global marketing campaigns. A more detailed contract also stabilises labour relations. The terms and conditions of work are clearly established and opportunities for ‘off-piste’ negotiations are thus reduced. There is little concern that this may reduce the number of potential surrogates, for as the Director of one clinic explained to us, they are so oversubscribed with prospective surrogates that they employ only one in every ten who applies.

Second, contract effectively displaces attention from the real loci of negotiation. A contract does not pertain until the actual surrogacy is well underway and it is, moreover, based on the presumption that only three parties are involved: the clinic, the surrogate or donor, and the intending parents. However, as I have noted earlier, these labourers are not recruited directly by the clinic. They are inducted into this work through a complex and multifaceted network of kin relations and their continuance in this occupation is shaped by a variety of relations, exchanges and transactions—material, emotional and psychic—that operate ‘extra legally’ and which thus, cannot be effectively ‘contractualised’. These include, for example, gifts of food, medication, transport and monetary advances that arise out of the relationships built between the surrogate or donor and their agent, between the agent and the hospital staff, and the staff and surrogate themselves in a complex affective matrix.

This gifting alters the intrinsic dynamics of this economy ultimately rendering contracts largely moot for surrogates and donors. It does so by decentring the formal labour relations that are foregrounded in the contract with something more closely allied to the kin relations with which these women are much more familiar. The vast majority of them have no experience of formalised labour or of unionisation and they remain in relationships of acute dependency with their agents. These are the individuals to whom they must appeal in negotiations about conditions of work or recompense. In fact one of the glaring deficiencies of current contracts is that they completely omit any reference to the agent or other intermediaries who actually broker the arrangements. The surrogates or donors cannot be said to have any kind of independent relationship with the clinic or hospital to whom they are contracted, moreover, they do not have any of the educational resources required to read contracts or to understand legalese. The only corporate entity to which they could appeal in the case of a breach of contract is the hospital, however, for the reasons outlined above they would never venture to undertake this themselves. Redress here remains something to be negotiated delicately through a web of personal relationships that will sustain and shape the surrogate's life prospects long after the terms of the formal contract have expired.

## Nationalisation and transnationalisation

A last set of presumptions that underpin recent analyses of the neoliberalisation of reproductive services are those surrounding its social and spatial dynamics. Advances in cryogenic preservation have enabled gametes to be circulated internationally creating a genuinely global market for such resources while new communication, transport and biotechnologies coupled with the rapid professionalisation of service provision in emerging economies have made transnational access to care both viable and affordable.^30^ It is routinely argued that this new bioeconomy in reproductive labour and services is organised ‘according to a map of regional and global economic power relations’ which, as Cooper and Waldby argue, ‘itself maps onto older histories of race and empire’.^31^ In such imaginings it is the (probably coloured) subaltern reproductive workers of the world located in its most deprived localities who labour to realise the desires of a (probably white) and privileged class of parents who commission their work from the metropolitan centres of the West—a notion no doubt cemented by many published reports and journalistic accounts that place inordinate emphases on just such dynamics. While this affords a generalised analysis of the broad contours of this trade it fails to capture significant discontinuities and variations in practice.

Much has been made of the fact that parents who commission surrogacy in India exploit the racial or ethnic characteristics of this class of reproductive labourers, selecting as donors those whose fair skin colour accords most closely to their own, while simultaneously relegating women with darker skin tones to the role of gestational carrier thereby hierarchising the value of their respective genetic ‘stocks’. However, as I have argued elsewhere, the practice of ‘qualifying’ gametes through use of a variety of metrics and devices such as genetic screening and donor profiling is a cornerstone of all reproductive banking worldwide. The resultant ‘pedigree’ is used as an important tool of selection for all those who seek a sperm donor from California Cryobank, for example, a cohort of consumers that includes a multitude of individuals located across the globe including in many developing countries.^20^ In an interesting reversal of the presumed polarity of this trade they also seek to ‘exploit’ the racial, genetic and educational attributes of the white highly professionalised donors recruited to such banks, the majority of which are located in the metropolitan centres of the global North. In this case the clinical labour of donor is being mobilised in precisely the opposite direction to that imagined by these narratives of neoliberalism with an attendant reversal of its presumed racial and geographical dynamics.

Our recent investigations in India have also revealed that the vast majority of those commissioning gestational surrogacies in the fertility clinics that we observed in in both Mumbai and Jaipur were not couples from the USA, Britain, Canada, Japan or Australia, nor were they non-resident Indians. In fact, most commissions came from India's very rapidly gentrifying middle and upper classes that now also have the financial resources necessary to ‘outsource’ their reproductive needs. This kind of surrogacy is becoming, according to one of the Director's of such a clinic, ‘entirely normalised’. This suggests that India's engagement with reproductive technologies is now highly differentiated with many Indian woman of these classes internalising and acting on the socially and racially stratified divisions of labour that characterise post-Fordist work/life (wheresoever encountered), thus becoming themselves active consumers of other's reproductive service labour.

Lastly, and relatedly, it is often assumed that the increase in India's infertility service capacity has arisen to meet international demand for outsourced reproductive services as an artefact of a general neoliberalisation of provision. From this perspective emerging economies are again characterised as occupying a subordinate position in the supply chain. However, as the Chief Executive Officer of one of India's leading infertility supply companies recently explained to me, it is India's leading in vitro fertilisation specialists who have constructed the most extensive networks of fertility clinics in the developing world building their own ‘empires’ of commercialised reproductive care in countries from Africa to Thailand and the Gulf States.

## Conclusion

Much current research on the emergence of forms of reproductive labour such as gamete donation and GS characterise this as a form of ‘clinical labour’, the outsourcing and contractualisation of which, are seen as artefacts of a wider neoliberalisation of the service economy. While such accounts provide a compelling overarching narrative of the ways in which the dynamics of reproduction have been reordered by their subjugation to capital they only partially capture the profound variations in labour relations that attend these practices as they are performed in highly differentiated localities across the world. Any attempt to undertake assessments of their exploitative potential or to arrive at normative positions on regulation must necessarily be informed by detailed ethnographic research that elucidates the complex lived experience of clinical labour in situ. In so doing such analyses must necessarily also attend to the question of how power relations within the neoliberal economy are shaped by longer histories of unevenness and geopolitical and social in equality. In critically engaging with such accounts this article hopefully has demonstrated just some of other ways in which these narratives of neoliberalism are complicated in practice and with what effects for our theorisations of clinical labour.

## Footnotes

i There now exists a large literature on gestational surrogacy and cross-border reproductive care. For a summation of that literature please see reference 4.

ii Carolin Schurr of the University Of Zurich who is currently undertaking a five year Branco-Weiss Fellowship into the transnationalisation of markets in reproductive services has reported to me anecdotally a considerable increase in traffic to Mexico's fertility clinics post the baby Gammy furore. A high proportion of this traffic is comprised of gay couples who have been excluded from India and Thailand following the introduction of regulations banning their access to surrogacy.

iii These include two reports from the Permanent Bureau of the Hague Conference on Private International Law: Private International Law Issues Surrounding the Status of Children, including Issues Arising from International Surrogacy Arrangements, Preliminary Document No. 11 (March 2011) and A Preliminary Report on the Issues From International Surrogacy Arrangements, Preliminary Document No. 10 (March 2012) (noting growth of nearly 1,000% in the market for such arrangements between 2006 and 2010).

iv This study entitled “Reproductive Ethics in Context: The Indian Experience” is a Wellcome Trust Funded International Bioethics Project Grant Number: 097733/B/11/2 is due for completion in 2016. To date, interviews have been conducted with 89 research participants including commissioning parents (domestic and transnational), regulators, surrogates, gamete donors, policy makers and industry representatives. Interviews have been conducted in Mumbai, Jaipur and in Delhi.

v To simply ensuring payments are received on time, for example.

vi This stands in stark contrast to the experience of another class of clinical labourers clinical trial recruits in the US who have been reportedly oft denied similar forms of non-contractual support. See reference 23.

vii Rising a reported 20% between 2010 and 2012, see reference 25.

viii Indian's Draft Assisted Reproductive Technologies (Regulation)Bill (2010) has been awaiting ratification for four years. It has been subject to continued debate and redrafting as new contingencies arise.
